# Penostatin Derivatives, a Novel Kind of Protein Phosphatase 1B Inhibitors Isolated from Solid Cultures of the Entomogenous Fungus *Isaria tenuipes*

**DOI:** 10.3390/molecules19021663

**Published:** 2014-01-29

**Authors:** Yu-Peng Chen, Chun-Gui Yang, Pei-Yao Wei, Lin Li, Du-Qiang Luo, Zhi-Hui Zheng, Xin-Hua Lu

**Affiliations:** 1 College of Life Science, Key Laboratory of Medicinal Chemistry and Molecular Diagnosis of Ministry of Education, Hebei University, Baoding 071002, China; 2 Biotechnology Center of Hebei Province, Hebei University, Baoding 071002, China; 3 New Drug Research and Development Center, North China Pharmaceutical Group Corporation, Shijiazhuang 051007, China

**Keywords:** *Isaria tenuipes*, penostatin derivatives, protein phosphatase 1B inhibitor

## Abstract

Protein tyrosine phosphatase 1B (PTP1B) is implicated as a negative regulator of insulin receptor (IR) signaling and a potential drug target for the treatment of type II diabetes and other associated metabolic syndromes. Therefore, small molecular inhibitors of PTP1B can be considered as an attractive approach for the design of new therapeutic agents of type II diabetes diseases. In a continuing search for new protein phosphatase inhibitors from fungi, we have isolated a new compound, named penostatin J (**1**), together with three known ones, penostatin C (**2**), penostatin A (**3**), and penostatin B (**4**), from cultures of the entomogenous fungus *Isaria tenuipes.* The structure of penostatin J (**1**) was elucidated by extensive spectroscopic analysis. We also demonstrate for the first time that penostatin derivatives exhibit the best PTP1B inhibitory action. These findings suggest that penostatin derivatives are a potential novel kind of PTP1B inhibitors.

## 1. Introduction

Tyrosine phosphorylation and dephosphorylation are crucial elements in eukaryotic signal transduction [1]. Phosphatases can be subdivided into the protein tyrosine phosphatase (PTP) and protein serine/threonine phosphatase (PSP) classes [2]. The PTP superfamily are divided into more than three families: (i) ‘classical PTPs’, which exist both as transmembrane forms (such as LAR or PTP-a) and non-transmembrane forms (such as PTP1B or TC-PTP); (ii) the dual-specificity phosphatases, which are able to dephosphorylate both phosphotyrosine and phosphothreonine in specific sequence contexts; (iii) the low molecular weight phosphotyrosine protein phosphatases (LMW-PTPs) [3,4]. More recent evidence has suggested PTP1B as a major negative regulator of the insulin signaling pathway [5,6]. As so far, several ‘classical’ PTP are attractive therapeutic targets, including PTP1B for obesity and type II diabetes; SHP2 for cancer and Lyp for rheumatoid arthritis [6]. Although the research efforts were made in academia and industry over the past decade, there are very few PTPase inhibitors that have been advanced into clinical trials [7]. Thus, inhibitors of PTP1B can also be considered as an attractive approach for the design of new therapeutic agents for the treatment of type II diabetes and new antitumoral drugs. To date very few inhibitors have isolated from microorganisms, particularly insect pathogenic fungi [8]. Therefore, insect pathogenic fungi have been considered as an untapped source of small molecules PTP inhibitors.

**Figure 1:**
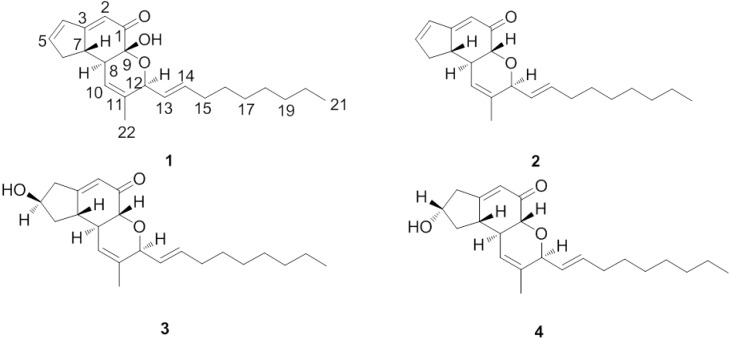
The structures of compounds **1**–**4**.

Insect pathogenic fungi are an ecologically highly specialized group of microorganisms. Some 700 entomopathogenic species are presently known, belonging to the families of Deuteromycetes, Ascomycetes, Zygomycetes, Oomycetes, Chytridiomycetes, Trichomycetes and Basidiomycetes [8]. Insect pathogenic fungi produce a plethora of insecticidally and pharmaceutically active compounds. For example, the cyclic depsipeptides destruxins [9], beauvericin, bassianolides, the beauveriolides [10] and lactams, such as pyridovericin [11] have been reported from some Deuteromycetes. Especially at 2010, fingolimod (Gilenya, FTY720), a synthetic compound based on the insect fungal secondary metabolite myriocin (ISP-I) [12], is a potent immunosuppressant that was approved (September 2010) by the U.S. FDA as a new treatment for multiple sclerosis (MS) [13]. Therefore, interest in searching for bioactive compounds from insect pathogenic fungi has increased considerably recently [14,15]. China has been proven to be a rich source of insect pathogenic fungi [16], this prompted us to undertake further phytochemical investigation. In a continuing search for new PTP1B inhibitors from insect pathogenic fungi, we have isolated a new compound, named penostatin J (**1**), together with the known ones penostatin C (**2**), penostatin A (**3**), and penostatin B (**4**) (Figure 1) from cultures of the entomogenous fungi *Isaria tenuipes.* Details of the isolation and structural elucidation of **1** are reported herein.

## 2. Results and Discussion

**Figure 2:**
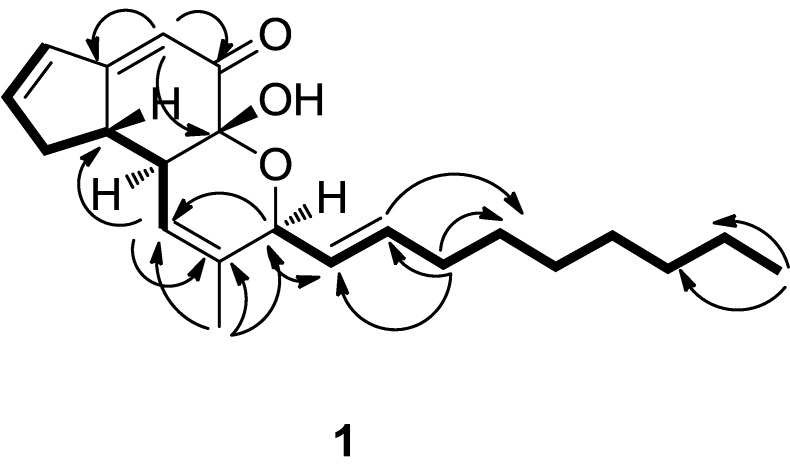
The ^1^H-^1^H-COSY, selected key HMBC correlations of **1**.

Compound **1** was obtained as a yellow oil. The HRESI-MS of **1** gave an [M+Na]^+^ peak at *m/z* 365.20820 (calcd for C_22_H_30_NaO_3_, 365.20872) and corresponded to a molecular formula of C_22_H_30_O_3_, requiring eight degrees of unsaturation. A close inspection of the ^1^H- and ^13^C-NMR data (Table 1) of **1** by DEPT techniques and HMQC revealed the presence of a conjugated ketone at *δ*(C) 194.7, two disubstituted double bonds at *δ*(C) 128.1 and 135.6, and 131.4 and 150.9, two trisubstituted double bonds (four olefinic carbons at δ (C) 113.8 and 174.6, and 119.7 and 134.5), an allylic methyl at *δ*(C) 18.2, a primary methyl at *δ*(C) 12.9, seven methylenes (C6 and C15–C20) and three sp^3^-hybridized methines (C7, C8, and C12) including one oxygen-bearing methine. An analysis of the ^1^H and ^13^C-NMR spectral data (Table 1) indicated compound **1** was very similar to penostatin C, which suggested that compound **1** possesses the same substitution pattern [17]. The distinct difference between **1** and **2** are that: the chemical shifts value at C-9 of **1** (*δ_C_* 91,9 (s)) were absent in **2** (*δ_C_* 74.9 (d)) which implied that the hydrogen of C-9 in **2** is replaced by hydroxy into a quaternary carbon in **1**. In the light of the evidences mentioned above and key ^1^H-^1^H COSY and HMBC correlations (Figure 2), the planar structure of **1** was thus elucidated as show in Figure 1. The stereochemistry for **1** was established by comparison observed coupling constants and NOESY data with penostatins A–D [17]. The relative configuration of C-7, C-8 and C-12 except for C-9 was shown to be the same as that of **2** by the coupling constants of H-8 to H-7, and NOEs from H-22 to H-12, H-13 and H-14. This was also supported by the measurement of J12,13 (6.5 Hz) and J13,14 (15.5 Hz) coupling constants in **1** which were the same as in **2**. The absolute configuration of C-9 was further determined by comparing the circular dichroism (CD) and [α]_D_ spectra with compound **2 (**Supporting Information**). **The positive Cotton effect at 201 nm in the CD spectrum of **1** indicated the 9*R* configuration, supporting the abovementioned absolute stereostructure for **1**. In addition**, **the Cotton effect at 289 nm, which is considered to correspond to that at 274 nm in **2**, was found as a negative sign as in **2**. This result supported the 7S configuration of **1** [18]. Finally, on the basis of these data, the stereochemistry of **1** was thus determined as shown (Figure 1) and the compound was named penostatin J.

### PTP1B Activities

Compounds **1**–**4** displayed significant PTP1B inhibitory action with IC_50 _ values from 0.37 to 43.6 μM (Table 2). It is important to note that compound **1** and **2** have exhibited significant selectivity between PTP1B and LAR. LAR exists as a transmembrane form, but LTP1Bs exist as a non-transmembrane forms. To our knowledge, the PTP1B inhibitory action of compounds **1** and **2** is the same as that of ertiprotafib and MSI-1436, two compounds with IC_50_ values of 1.6 and 1.0 μM, respectively, whose inhibitory action has been evaluated *in vivo* (Phase II and I) [19]. This is first time that penostatin derivatives are reported to have significant PTP1B inhibitory action.

**Table 1:** ^1^H and ^13^C-NMR data for **1** and penostatin C (**2**).

Position	1		Penostatin C (2)	
	*δ*(H)	*δ*(C)	*δ*(H)	*δ*(C)
1		194.7		197.8
2	5.95 (d, *J* = 2.4 Hz)	113.8	5.87 (d, *J =* 2.4 Hz)	115.7
3		174.6		173.8
4	6.54 (dt, *J =* 5.6; 2.3 Hz)	131.4	6.52 (dt, *J =* 5.5; 2.4 Hz)	131.6
5	6.87 (dt, *J =* 5.6; 2.3 Hz)	150.9	6.82 (dt, *J =* 5.5; 2.3 Hz)	149.4
6	2.90 (dddd, *J =* 16.9; 5.9; 1.5; 2.0 Hz)	36.8	2.89 (dddd, *J =* 17.5; 6.5; 1.5; 2.0 Hz)	36.2
	2.52 (dddd, *J =* 16.9; 4.1; 1.5; 2.0 Hz)		2.50 (dddd, *J =* 17.5; 4.2; 1.5; 2.0 Hz)	
7	2.99 (dddd, *J =* 10.8; 7; 3.7; 2.3 Hz)	46.5	2.77 (dddd, *J =* 11.2; 6.8; 3.6; 2.5 Hz)	45.7
8	2.28 (tq, *J =* 10.8; 1.9 Hz)	44.9	2.49 (tq, *J =* 11.4; 2.2 Hz)	44.9
9		91.9	4.44 (d, *J =* 11.1 Hz)	74.9
10	5.76 (m)	119.7	5.67 (m)	115.7
11		134.5		135.9
12	4.67 (d, *J =* 6.5 Hz)	76.2	4.57 (d, *J =* 6.4 Hz)	77.7
13	5.37 (dd, *J =* 6.5; 15.5 Hz)	128.1	5.62 (dd, *J =* 6.6; 15.5 Hz)	126.3
14	5.79 (dd, *J =* 15.5, 6.5 Hz)	135.6	5.74 (dt, *J =* 15.5; 6.9 Hz)	136.2
15	2.08 (m)	31.8	2.09 (m)	31.8
16	1.42 (m)	28.7	1.31 (m)	28.6
17	1.31 (brs)	28.8	1.28 (brs)	28.7
18	1.31 (brs)	28.7	1.28 (brs)	28.6
19	1.31 (brs)	31.6	1.28 (brs)	31.6
20	1.31 (brs)	22.3	1.28 (brs)	22.3
21	0.91(t, *J =* 6.8 Hz)	12.9	0.88(t, *J =* 6.8 Hz)	13.0
22	1.65 (s)	18.2	1.59 (s)	18.7

Note: **1** and **2** were measured in CDCl_3_. Assignments made on the basis of ^1^H, ^1^H-COSY, HMQC and HMBC experiments.

**Table 2:** Potency and selectivity of penostatin derivatives PTP1B inhibitors.

Compound	IC_50_ (μM)	
	PTP1B	LAR ^a^
**1**	12.53 ± 0.9	a
**2**	0.37 ± 0.05	53.33 ± 4.06
**3**	15.87 ± 2.49	a
**4**	33.65 ± 7.33	a
**Sodium orthovanadate (positive control)**	0.65 ± 0.08	0.89 ± 0.02

Note: ^a^ represents no inhibition at 50 μg/mL. The data were expressed as mean ± SD of three replicates.

## 3. Experimental

### 3.1. General

Optical rotations were obtained on a Perkin-Elmer 341 spectropolarimeter. UV Spectra were recorded on a UV-210 spectrometer. IR spectra were recorded on a Perkin-Elmer 577 spectrometer. The ^1^H- and ^13^C-NMR spectra were recorded on a Bruker AM-600 spectrometer at 600 and 150.9 MHz using TMS as internal standard. HRESIMS data were measured on a Bruker FT-ICR-MS mass spectrometer. TLC was carried out using glass-precoated silica gel GF_254_ (Yantai Zhi Fu Chemical Co., Ltd, Yantai, China) and visualized under UV light or by spraying with vanilin (contains H_2_SO_4_) ethanol reagent. Sephadex LH-20 gel (25~100 μm, GE Healthcare Co., Ltd., Uppsala, Sweden), silica gel (200–300 mesh, Yantai Zhi Fu Chemical Co., Ltd) were used for column chromatography (CC).

### 3.2. Fungal Material and Cultivation Conditions

*Isaria* *tenuipes* was isolated from an unidentified lepidopteran collected in Hebei Province, China, and identified by Yong-Chun Niu, It has been catalogued as strain RCEF 37776 in the culture collection of the Key Laboratory of Medicinal Chemistry and Molecular Diagnosis of Ministry of Education, Hebei University. The fungal strain was cultured on slants of potato dextrose agar (PDA) at 28 °C for 7 d, and then inoculated into 500-mL Erlenmeyer flask containing 100 mL of PDA medium (20.0 g of glucose, 200.0 g of potato (peeled), 3.0 g of KH_2_PO_4_, 1.5 g of MgSO_4_, 0.1 g of citric acid, and 10.0 mg of thiamin hydrochloride, in 1 L of deionized H_2_O). The final pH of the media was adjusted to 6.5 before sterilization. After 7 d of incubation at 28 °C on rotary shakers at 150 rpm, mycelial pieces were transferred to 50 Erlenmeyer flask (500 mL) containing 200.0 g rice medium, (88 g rice, 110 mL distilled water) and the fermentation was carried out on a incubator for 40 d.

### 3.3. Extraction and Isolation

At the end of the incubation period, the mycelium and medium were extracted with EtOAc (20 L). The extract was concentrated to dryness and the residue (87.0 g) was separated on a silica gel column eluted with petroleum ether (PE)/EtOAc (100:0, 98:2, 95:5, 90:10, 60:10, 30:10, 10:10 (*v/v*)) to afford seven fractions, Frs.1–7. Fr.3 (500 mg) was additionally purified by CC eluted with PE/acetone 50:1 (*v/v*) and LH-20 (methanol) to afford compound **2** (6 mg; TLC (PE/EtOAc (45:1); R*_f_* = 0.6). Fr. 6 (3.0 g) eluted with PE/EtOAc (30:10) was repeatedly purified by CC (silica gel; PE/acetone 3:1 (*v/v*)) and Sephadex LH-20 (methanol) to obtain compound **1** (4.5 mg; TLC (CHCl_3_/MeOH 10:1); R_f_ = 0.6), **3** (7 mg; TLC (CHCl_3_/MeOH 10:1); *R_f_* = 0.5) and **4** (8 mg; TLC (CHCl_3_/MeOH 10:1); *R_f_* = 0.6), respectively.

### 3.4. Physico-Chemical Properties

*Penostatin J* (**1**): Isolated as a pale yellow oil, 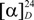 = 59.3 (c = 0.003, MeOH). IR (KBr) v_max_: 3442, 2926, 2854, 1659, 1618 cm^−1^. UV (CHCl_3_) λ_max_ (lg ε): 289 (3.45), 203 (3.35) nm. ^1^H- and ^13^C-NMR, see Table 1. Positive ion HR-ESI-MS [M+Na]^+^ *m/z* 365.20820 (calcd for C_22_H_30_O_3_Na, 365.20872).

*Penostatin C* (**2**): Isolated as a yellow oil. 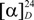 = 87.5 (c = 2.2, MeOH). IR (KBr) v_max_: 3427, 2926, 2854, 1658, 1618 cm^−1^. UV (CHCl_3_) λ_max_ (lg ε): 280 (3.39), 0.3422 (3.54) nm. ^1^H- and ^13^C-NMR, see Table 1. Positive ion HR-ESI-MS [M+Na]^+^ *m/z* 349.22453 (calcd for C_22_H_30_O_2_Na, 349.22445). It was identified as penostatin C by comparison of the spectral data with the literature [17,18].

*Penostatin A* (**3**): Isolated as a yellow oil. 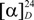 = +56.8 (c = 2.2, MeOH). IR (KBr) v_max_: 3425, 2924, 2853, 1675, 1633 cm^−1^. UV (CHCl_3_) λ_max_ (lg ε): 253 (3.78), 202 (3.90) nm. ^1^H-NMR (CDCl_3_) δ 5.64 (1H, s, H-2), 2.42 (1H, d, *J =* 1.74 Hz, H-4α), 2.98 (1H, dd, *J =* 7.5 Hz, 18.5, H-4β), 4.43 ((1H, m, H-5), 2.53 (1H, ddd, *J =* 11.8,7.0,1.9 Hz, H-6α), 1.50 (1H, td, *J =* 12.5,4.8 Hz, H-6β), 2.45 (1H, m, H-7), 2.53 (1H, m, H-8), 4.08 (1H, d, *J =* 11.5 Hz, H-9), 5.92 (1H, m, H-10), 4.55 (1H, d, *J =* 6.5 Hz, H-12), 5.62 (1H, dd, *J =* 13.9,5.6 Hz, H-13), 5.74(1H, dt, *J =* 13.8,6.7 Hz, H-14), 2.12 (2H, m, H-15), 1.42 (2H, m, H-16), 1.31 (4H, brs, H-17, 18, 19, 20), 0.91 (3H, t, *J =* 6.8 Hz, H-21), 1.69 (3H, s, H-22). ^13^C-NMR (CDCl_3_) δ 197.1 (s, C-1), 121.5 (d, C-2), 172.3 (s, C- 3), 40.9 (t, C-4), 70.2 (d, C-5), 37.8 (t, C-6), 45.4 (d, C-7), 44.9 (d, C-8), 73.7 (d, C-9), 121.2 (d, C-10), 135.9 (s, C-11), 77.5 (d, C-12), 126.3 (d, C-13), 135.8 (d, C-14), 31.8 (t, C-15), 28.7 (t, C-16), 28.7 (t, C-17), 28.7 (t, C-18), 31.6 (t, C-19), 22.3 (t, C-20), 13.0 (q, C-21), 18.7 (q, C-22). Positive ion HR-ESI-MS [M+Na]^+^ *m/z* 367.23543 (calcd for C_22_H_32_O_3_Na, 367.23514). It was identified as penostatin A by comparison of the spectral data with the literature [17,18].

*Penostatin B* (**4**): Isolated as a yellow oil. 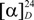 = −24.5 (c = 2.2, MeOH). IR (KBr) v_max_: 3460, 2921, 1671, 1632, 1466 cm^−1^. UV (CHCl_3_) λ_max_ (lg ε): 234 (3.43), 202 (3.45) nm. ^1^H-NMR (CDCl_3_) δ 5.65 (1H, s, H-2), 2.36 (1H, t, *J =* 1.68 Hz, H-4α), 2.87 (1H, m, H-4β), 4.50 (1H, m, H-5), 2.63 (1H, d, *J =* 19.8 Hz, H-6α), 1.57 (1H, ddd, *J =* 10.7, 9.2, 5.9 Hz, H-6β), 2.27 (1H, m, H-7), 2.85 (1H, m, H-8), 4.14 (1H, *d*, *J* = 11.6 Hz, H-9), 5.93 (1H, m, H-10), 4.57 (1H, d, *J =* 6.5 Hz, H-12), 5.62 (1H, dd, *J =* 5.5, 6.0 Hz, H-13), 5.74(1H, dt, *J =* 13.8, 6.7 Hz, H-14), 2.12 (2H, m, H-15), 1.42 (2H, m, H-16), 1.31 (4H, brs, H-17, 18, 19, 20), 0.91 (3H, t, *J =* 6.8 Hz, H-21), 1.69 (3H, s, H-22). ^13^C-NMR (CDCl_3_) δ 198.3 (s, C-1), 122.9 (d, C-2), 174.5 (s, C-3), 42.4 (t, C-4), 71.4 (d, C-5), 39.5 (t, C-6), 46.2 (d, C-7), 46.1 (d, C-8), 75.1 (d, C-9), 122.4 (d, C-10), 137.3 (s, C-11), 78.8 (d, C-12), 127.5 (d, C-13), 137.1 (d, C-14), 33.1 (t, C-15), 29.9 (t, C-16), 29.9 (t, C-17), 30.0 (t, C-18), 32.9 (t, C-19), 23.5 (t, C-20), 14.3 (q, C-21), 19.9 (C-22). Positive ion HR-ESI-MS [M+Na]^+^ *m/z* 367.23523 (calcd for C_22_H_32_O_3_Na, 367.23514). It was identified as penostatin B by comparison of the spectral data with the literature [17,18].

### 3.5. Biological Evaluation

PTP1B activity was measured as the rate of hydrolysis of *p*-nitrophenyl phosphate (*p*NPP) in a 96-well microtiter plate format [20]. Standard assays were conducted at room temperature in a total volume of 0.2 mL that contained HEPES buffer (50 mM, pH 7.2), NaCl (50 mM), EDTA (1 mM), DTT (1 mM), bovine serum albumin (1 mg/mL), *p*NPP (at various concentrations, Km) 1.4 (0.03 mM), and PTP1B (35 ng/mL). Sodium orthovanadate was used as the positive control. Inhibitors were added in DMSO at 100 times the final concentration. PTP1B activity was measured as the rate of inorganic phosphate released upon hydrolysis of the IR phosphopeptide, TRDIpYETDpYpYRK, using a Malachite Green method [21]. The enzyme activity was estimated by measuring the absorbance at 405 nm with appropriate corrections. Each experiment was performed either in triplicate, and IC_50_ data were derived from three independent experiments.

Activities of LAR were measured as described above except that the HEPES buffer (50 mM) was at pH 6.8, and the enzyme was added at a concentration of 10 μg/mL. Activity of calcineurin (from Sigma Chemical Co.) was assayed using 4-methylumbelliferyl phosphate (0.8 mM) as a substrate in an assay mixture that contained Tris-HCl buffer (40 mM, pH 8.6), NaCl (100 mM), CaCl_2_ (0.5 mM), DTT (0.5 mM), bovine serum albumin (0.1 mg/mL), calmodulin (50 nM), and calcineurin (25 nM). Sodium orthovanadate was used as the positive control. Fluorescence at 450 nm was monitored with excitation at 400 nm. The color was allowed to develop at room temperature for 30 min, and the sample absorbances were determined at 650 nm using a plate reader (Molecular Devices, Sunnyvale, CA, USA). Each experiment was performed either in triplicate, and IC_50_ data were derived from three independent experiments.

Calculations. PTPase activities, based on a potassium phosphate standard curve, were expressed as nanomoles of phosphate released/min/mg protein. Inhibition of recombinant h-PTP1B by test compounds was calculated as percent of phosphatase control. A four-parameter nonlinear logistic regression of PTPase activities using SAS release 6.08, PROC NLIN, was used for determining IC_50_ values of the test compounds. The reported IC_50_ values show significant fit to the regression curve (*p* < 0.05).

## 4. Conclusions

On the whole, we have isolated a new compound, named penostatin J (**1**), together with three known compounds penostatin C (**2**), penostatin A (**3**) and penostatin B (**4**), from cultures of the entomogenous fungus *Isaria tenuipes*. We also demonstrate for the first time that penostatin derivatives have the best PTP1B inhibitory action. These findings suggest the penostatin derivatives are a potential novel kind of PTP1B inhibitor.

## Supplementary Materials

Supplementary materials can be accessed at: http://www.mdpi.com/1420-3049/19/2/1663/s1.
