# Supplementary material for: Penostatin Derivatives, a Novel Kind of Protein Phosphatase 1B Inhibitors Isolated from Solid Cultures of the Entomogenous Fungus Isaria tenuipes
Source: Molecules. 2014 Jan 29;19(2):1663–71. doi: 10.3390/molecules19021663 (PMC6270892; doi:10.3390/molecules19021663)

Supplementary File

Figure S1. The circular dichroism (CD) spectrum of compound **1**. (**A**) CD spectrum of compound of **1**; (**B**) Absorbance curve of componund **1**.

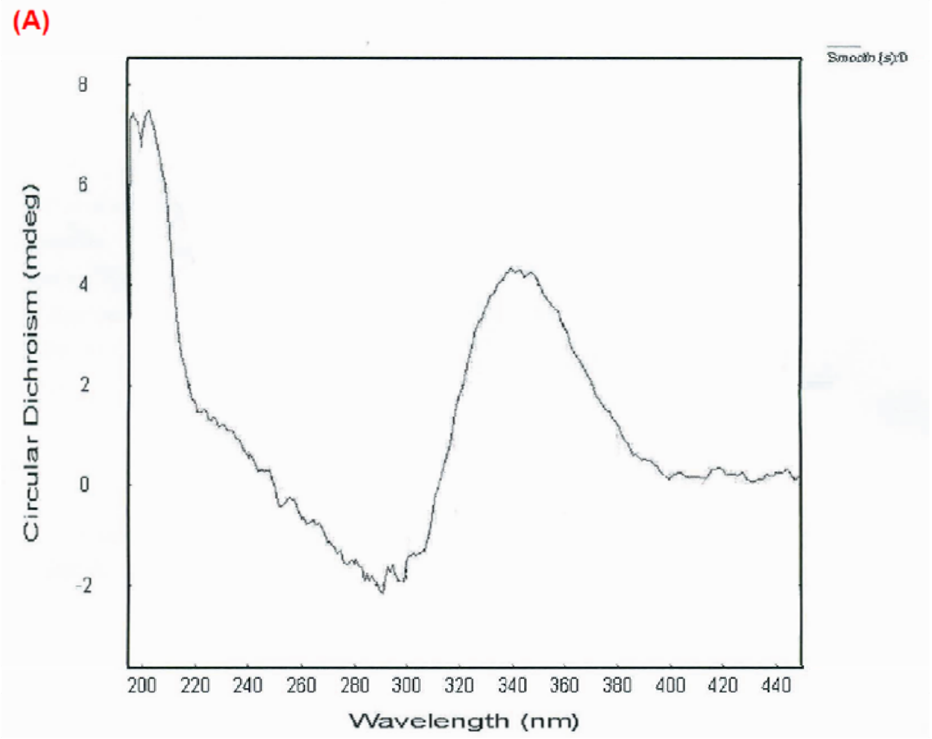

(**A**)

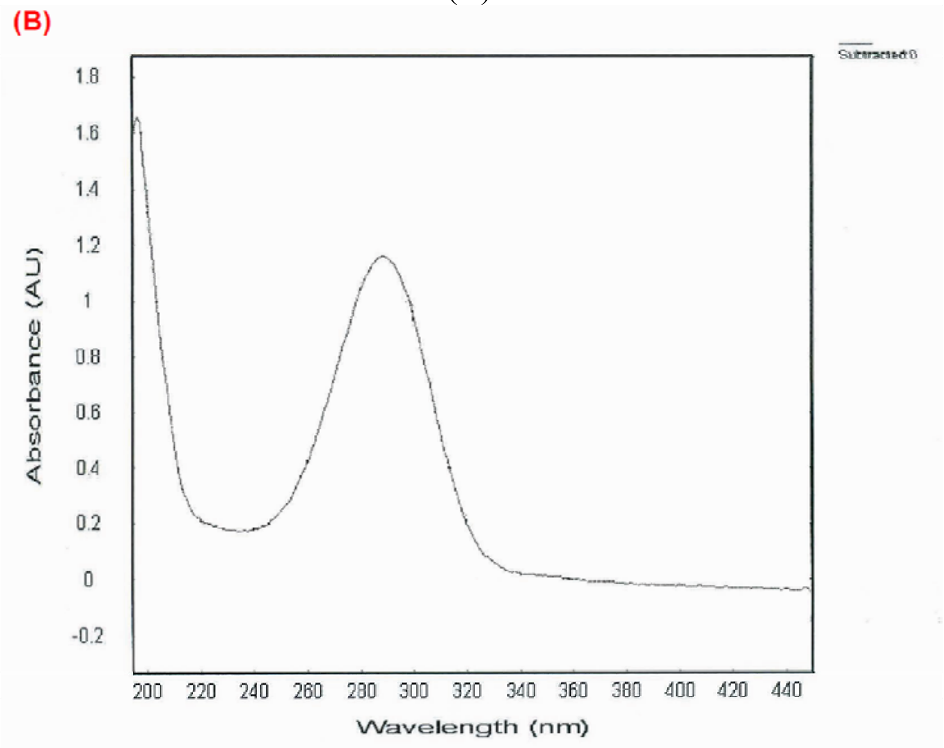

(**B**)

Figure S2. The [α]D spectrum of compound **1**.

Optical rotation measurement

Model : P-1020 (A060460638)

No.	Sample	Mode	Data	Monitor<br>Blank	Temp.<br>Cell<br>Temp Point	Date<br>Comment<br>Sample Name	Light<br>Filter<br>Operator	Cycle Time<br>Integ Time
No.1	4 (1/3)	Sp.Rot	59.3330	0.0178<br>0.0000	13.5<br>10.00<br>Cell	Mon Jan 20 15:26:48 2014<br>0.00300g/mL MeOH<br>YYCG-31	Na<br>589nm	2 sec<br>10 sec
No.2	4 (2/3)	Sp.Rot	59.3330	0.0178<br>0.0000	13.5<br>10.00<br>Cell	Mon Jan 20 15:27:01 2014<br>0.00300g/mL MeOH<br>YYCG-31	Na<br>589nm	2 sec<br>10 sec
No.3	4 (3/3)	Sp.Rot	59.3330	0.0178<br>0.0000	13.5<br>10.00<br>Cell	Mon Jan 20 15:27:15 2014<br>0.00300g/mL MeOH<br>YYCG-31	Na<br>589nm	2 sec<br>10 sec

Figure S3. The UV spectrum of compound **1**.

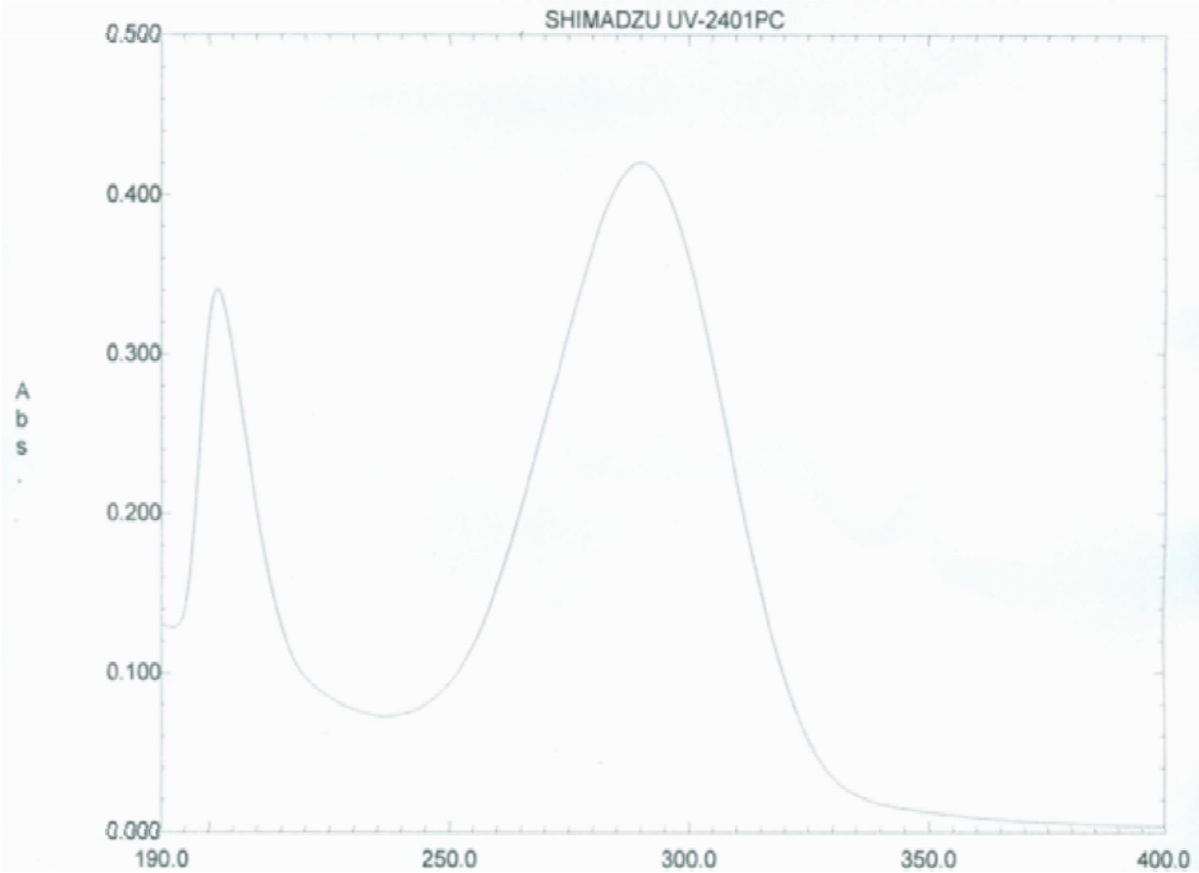

Figure S4. The HIESIMS spectrum of compound **1**.

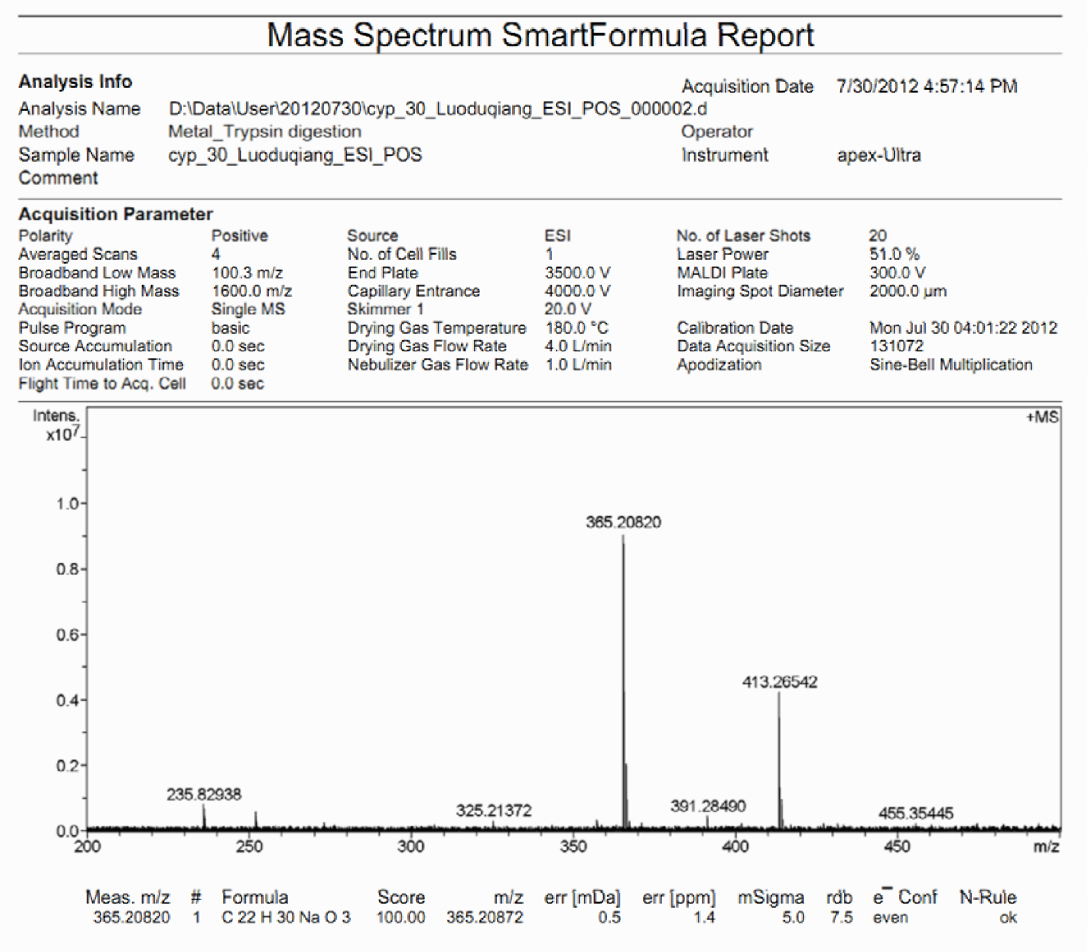

Figure S5. The ^{1}H-NMR spectrum of compound **1**.

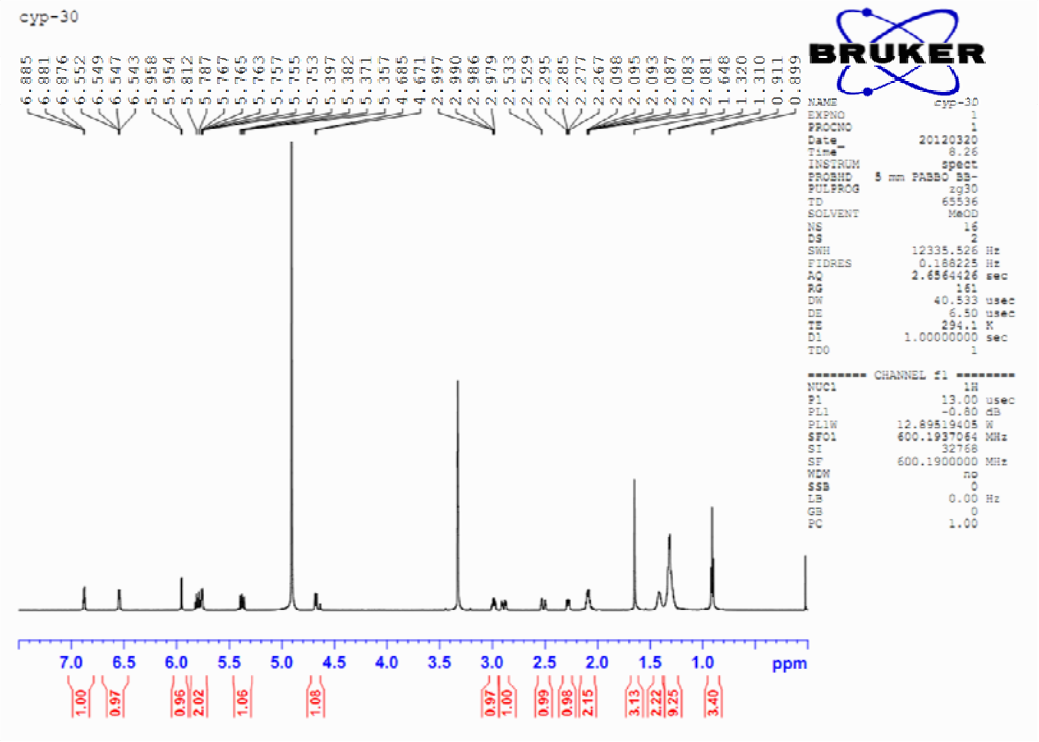

Figure S6. The ^{13}C-NMR spectrum of compound **1**.

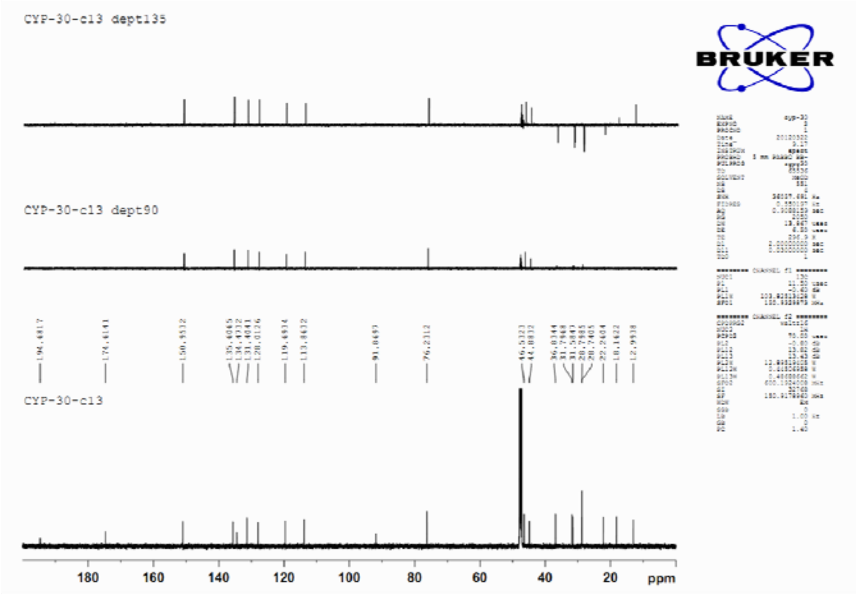

Figure S7. The HMQC spectrum of compound **1**.

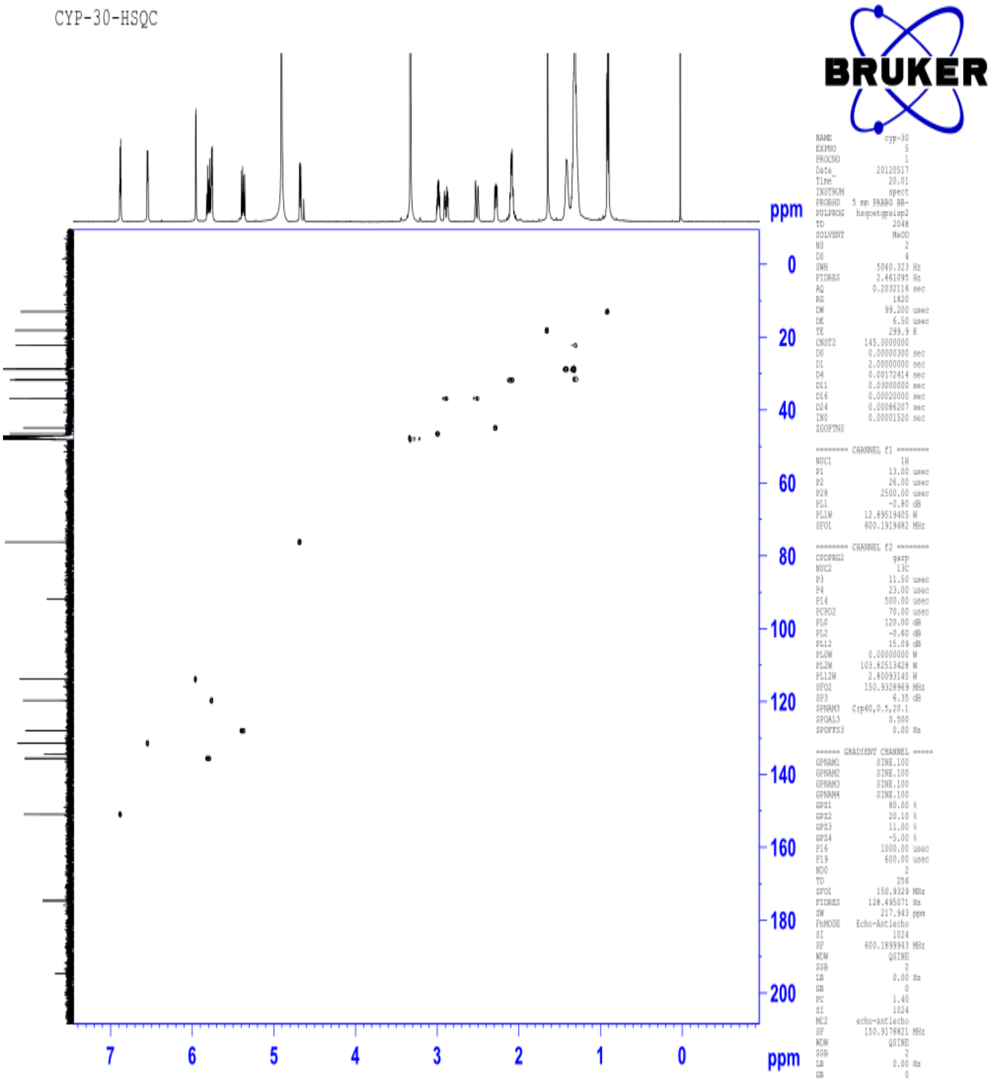

Figure S8. The ^{1}H-^{1}H COSY spectrum of compound **1**.

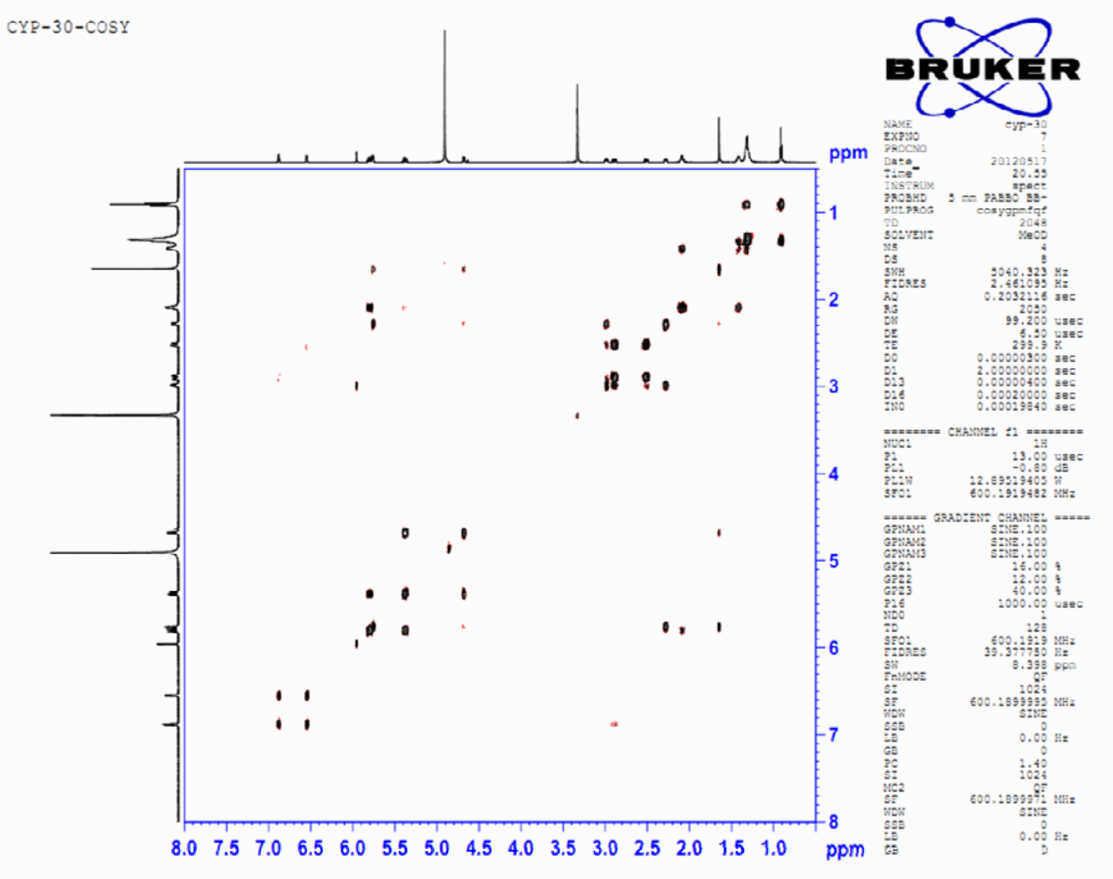

Figure S9. The HMBC spectrum of compound **1**.

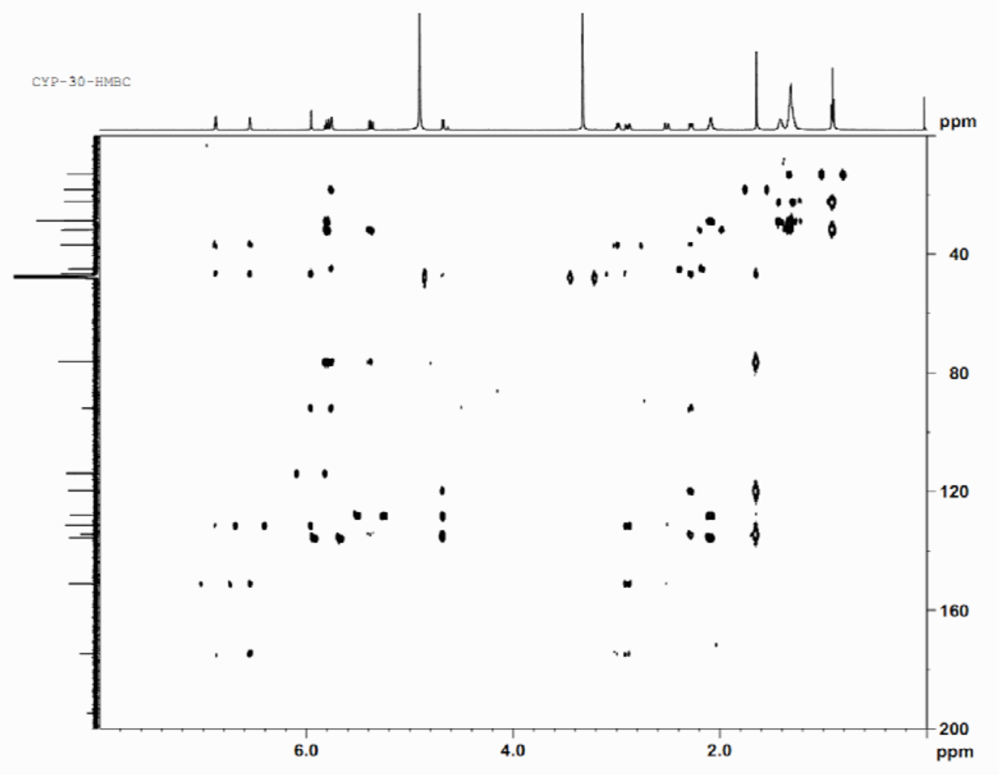

Figure S10. The ROESY spectrum of compound **1**.

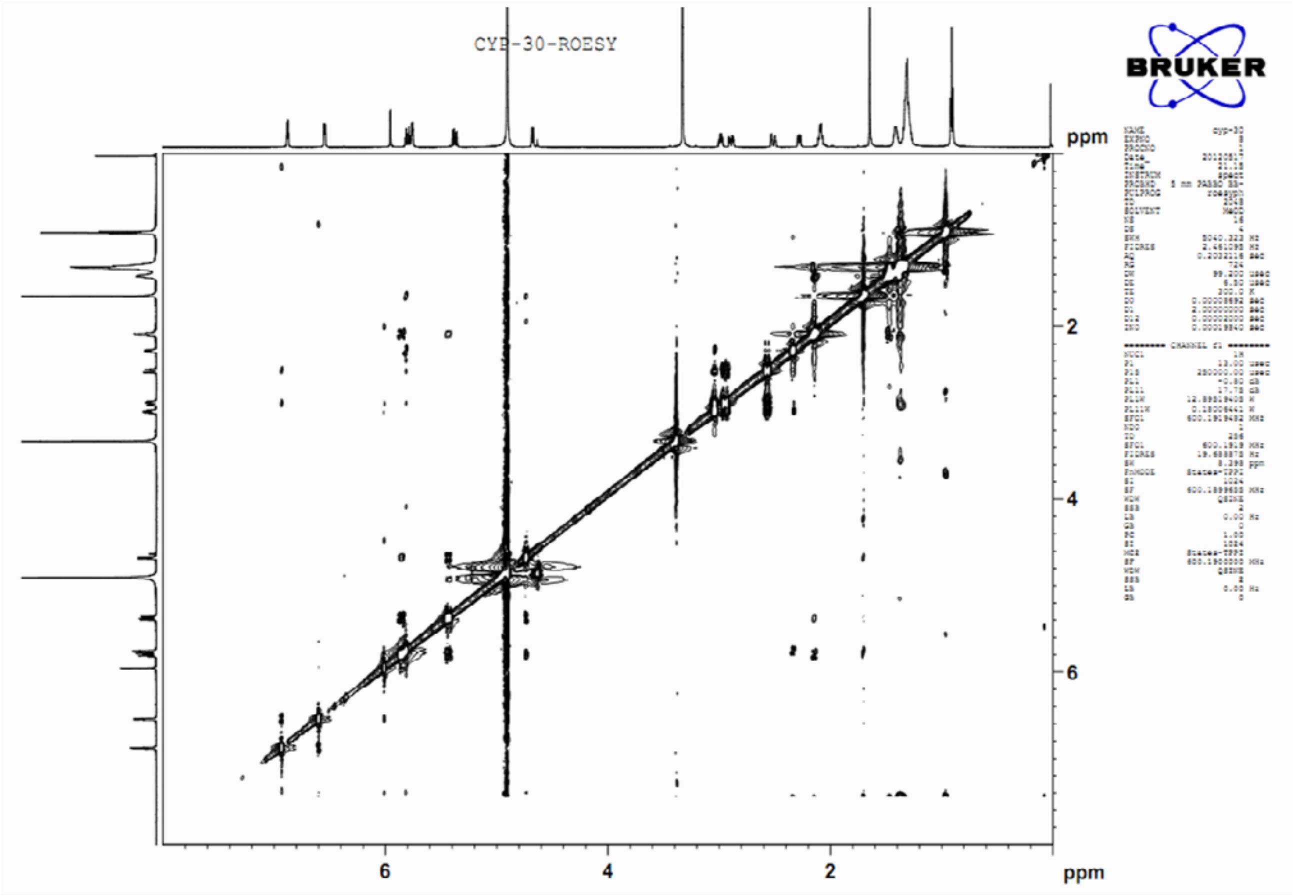

Supplement: Supplementary file 1 [file molecules-19-01663-s001.pdf]
